# Management of Warfarin Anticoagulation in Patients with Fractured Neck of Femur

**DOI:** 10.5402/2011/294628

**Published:** 2011-02-24

**Authors:** Feras Ashouri, Wissam Al-Jundi, Akash Patel, Jitendra Mangwani

**Affiliations:** ^1^Queen Alexandra Hospital, Portsmouth, UK; ^2^Northern General Hospital, Sheffield, UK; ^3^Colchester General Hospital, Colchester, UK; ^4^Great Western Hospital, Swindon, UK

## Abstract

*Background*. Most orthopaedic units do not have a policy for reversal of anticoagulation in patients with hip fractures. The aim of this study was to examine the current practice in a district general hospital and determine difference in the time to surgery, if any, with cessation of warfarin versus cessation and treatment with vitamin K. *Methods*. A retrospective review of the case notes between January 2005 and December 2008 identified 1797 patients with fracture neck of femur. Fifty seven (3.2%) patients were on warfarin at the time of admission. Patients were divided into 2 groups (A and B). Group A patients (16/57; 28%) were treated with cessation of warfarin only and group B patients (41; 72%) received pharmacological therapy in addition to stopping warfarin. Time to surgery between the two groups was compared. *Results*. The mean INR on admission was 2.9 (range 1.7–6.5) and prior to surgery 1.4 (range 1.0–2.1). Thirty eight patients received vitamin K only and 3 patients received fresh frozen plasma and vitamin K. The average time to surgery was 4.4 days in group A and 2.4 days in group B. The difference was statistically significant (*P* < .01). *Conclusion*. Reversal of high INR is important to avoid significant delay in surgery. There is a need for a national policy for reversing warfarin anticoagulation in patients with hip fractures requiring surgery. Vitamin K is safe and effective for anticoagulation reversal in hip fracture patients.

## 1. Introduction

Approximately 1% of the UK population is on oral anticoagulation with vitamin K antagonists [1]. Warfarin therapy is commonly administered to elderly patients for conditions such as atrial fibrillation, prosthetic heart valves, and thromboembolic disorders [2]. This population group is prone to osteoporotic fractures, such as fracture neck of femur. Current recommendations state that surgery for hip fractures following patient optimisation should be undertaken early, ideally within 24–48 hours [3–8]. This has created a challenge for orthopaedic surgeons as warfarin anticoagulation can cause significant delay in the surgical management of these patients [9]. Reversal of anticoagulation to prevent excessive bleeding at the time of hip surgery is required. This, however, carries a risk of thromboembolism that is further superimposed by immobility and hip surgery itself [10–12]. On the other hand, delaying surgery results in increased morbidity and mortality [7]. Complications of recumbency include pneumonia, pressure sores, muscle wasting, and urinary tract infections [13]. 

In order to reverse anticoagulation, one can “wait and watch” for INR to correct itself by stopping warfarin only, or actively reverse anticoagulation using pharmacologic means. These include vitamin K, fresh frozen plasma, clotting factor concentrates, or a combination of these [14]. Studies have shown that it may take up to 4 days for INR to reach acceptable levels for surgery [15]. There is a lack of local and national guidelines for reversal of anticoagulation in patients with a hip fracture [16, 17]. This study examines the current practice of perioperative management of anticoagulation in hip fracture patients in a district general hospital setting. 

## 2. Patients and Methods

We retrospectively reviewed case notes of patients who underwent surgery for fracture neck of femur between January 2005 and December 2008 at our unit. Patients on warfarin were identified and evaluated. The clinical indication for warfarin therapy, INR on admission and prior to surgery were documented. Patients were divided into two groups. Group A patients were treated with cessation of warfarin only, and group B patients received pharmacological therapy in addition to stopping warfarin. Time to surgery (excluding any other reason for delay) between the two groups was compared. Statistical analysis was done using one-way analysis of variance (ANOVA) test. 

## 3. Results

Between January 2005 and December 2008, 1797 patients with fracture neck of femur underwent hip surgery in our unit. Fifty-seven (3.2%) of these patients were on warfarin therapy at the time of admission. The mean age was 79.3 years (range 58–99), and 34 (59.6%) were females. The indications for warfarin treatment were as follows:

(i) atrial fibrillation (AF), 40 patients (70.1%)

(ii) thromboembolism, 12 patients (21.1%)

(iii) prosthetic heart valves, 5 patients (8.8%).

The mean INR on admission was 2.9 (range 1.7–6.5) and prior to surgery 1.4 (range 1.0–2.1). Thirty patients (52.6%) had an extracapsular fracture; 28 were treated with a dynamic hip screw (DHS) and 2 with an intramedullary hip screw (IMHS). Twenty-seven patients (47.4%) had intracapsular fracture neck of femur; 24 were treated with a hemiarthroplasty and 3 with cannulated screws. The method of anticoagulation reversal (watchful waiting or pharmacologic) was decided by the admitting doctor, the Orthopaedic Consultant, or following discussion with the Haematology Consultant. High INR was managed by watchful waiting in 16 (28.1%) and pharmacologic therapy in 41 (71.9%) patients. Despite cessation of warfarin in the watchful waiting group, INR initially increased in 5 patients (8.8%). In the pharmacologic reversal group, 38 patients received intravenous (IV) vitamin K (5–10 mg) only, and 3 patients received fresh frozen plasma (FFP) and IV vitamin K (5–10 mg). Three patients who received FFP and vitamin K had high INR values (5.2, 5.5, 6.5) on admission and were discussed with the Haematology Consultant. 

Reasons for surgical delay in warfarinised hip fracture patients, other than INR, were obtained from the notes. Seven patients in total had surgery delayed due to reasons other than high INR. These included medically unwell patients requiring optimization (4 patients); awaiting echocardiogram (1 patients); awaiting pacemaker check (1 patient); awaiting further imaging to confirm fracture (1 patient). These patients were excluded from our study. The average time to surgery from admission in patients not given pharmacologic treatment was 4.4 days (range 1–8 days). The average time to surgery from admission in patients given pharmacologic treatment was 2.4 days (range 1–5 days). The difference between the 2 groups is statistically significant (*P* < .01). 

## 4. Discussion

Most of the patients (71.9%) were managed using vitamin K and waiting for the INR to reach acceptable levels for anaesthesia and surgery. Warfarin was stopped on admission for all patients. A watch and wait policy was used for 16 patients (28.1%). The lack of standard protocol resulted in patients being given variable treatments to reverse anticoagulation. The decision was generally dependent upon the INR level, the indication for warfarin, and the urgency for surgery. 

An interesting finding in 5 of our patients in the “watch and wait” group was an increase in INR value one day after admission. This was also observed in a study investigating surgical delay in patients on warfarin [14]. The erratic increase in INR was thought to be secondary to posttraumatic stress responses, immobilization, and long periods of fasting in surgical patients [14]. The additive effect of these factors on the anticoagulation effect of warfarin has also been demonstrated in an experimental study [18].

The mean INR on admission for our patients was 2.9 (range 1.7–6.5) and prior to surgery 1.4 (range 1.0–2.1). There is a consensus that performing surgery in anticoagulated patients will increase the risk of intra- and post operative bleeding despite adequate surgical technique [19–21]. Current guidelines recommend an INR of less than 1.5 before any major surgery [22]. However, some surgeons and anaesthetists still undertook hip fracture surgery with a slightly higher level. Three patients were operated with INR > 1.5, and two of them received intraoperative FFP. INR of greater than 1.5 exposes surgical patients to increased risks of intra- and post operative bleeding complications [19, 23, 24]. In addition, the type of anaesthesia may be affected by anticoagulation. Bleeding and neurological dysfunction may occur during the insertion or removal of a spinal or epidural catheter in an anticoagulated patient [25–27]. Warfarin therapy is a contraindication for regional anaesthesia, such as spinal and epidural [12]. 

The thromboembolic risk after discontinuing warfarin should also be taken into consideration. This depends on the clinical indication for warfarin, the period the patient remains without anticoagulation therapy, the degree of anticoagulation reversal, and the type of surgical procedure undertaken [23, 24]. There are specific clinical indications that entail a high risk for thrombosis. These include mechanical prosthetic heart valves and chronic atrial fibrillation in association with other stroke risk factors. The high-risk group also includes patients with recent venous or arterial thromboembolism within the preceding 3 weeks [28]. Lower risk patients are those with AF without additional risk factors for stroke patients with deep vein thrombosis treated for more than six months after the acute event and patients with a hypercoagulable state without a thrombotic complication [28]. In the high-risk group, the absolute risk of thromboembolism in a 6–8-day perioperative period when warfarin is interrupted is approximately 0.3%. This corresponds to approximately 0.03% in the low-risk group [28]. 

The average time to surgery from admission in patients in the “watch and wait” group was 4.4 days (range 1–8 days). The average time to surgery from admission in patients given pharmacologic treatment was 2.4 days (range 1–5 days). The difference between the 2 groups is statistically significant (*P* < .01). These results have reinforced data obtained from previous studies [9, 13, 14, 17, 29–31]. The findings of this study revealed little evidence to support watchful waiting as the best strategy in preventing delay for semiurgent surgery. It is clear that pharmacologic reversal of warfarin anticoagulation facilitates earlier surgery in patients with hip fractures. It is costeffective, as it decreases hospital inpatient stay [9]. There were no side effects of pharmacologic reversal in our study. Risks of intravenous and subcutaneous vitamin K include anaphylactoid and cutaneous reactions respectively. Recent studies have recommended oral vitamin K for warfarin reversal [17, 29, 32, 33]. There is minimal difference in postsurgery rewarfarinisation time following low dose vitamin K administration in hip fracture patients compared to “watch and wait” patients. This indicates few discharge delays due to postsurgery rewarfarinisation [9, 29]. 

There are no clear guidelines in the literature for anticoagulation reversal in hip fracture patients. For elective hip operations, most surgeons adopt a “wait and watch policy.” Warfarin is generally discontinued 5 days prior to the elective procedure for INR to become subtherapeutic [34]. In emergencies, such as young patients with hip fractures, a rapid correction of INR is required. Available options include vitamin K, infusion of fresh frozen plasma, or prothrombin complex concentrate. This depends on the INR level and the urgency of surgical intervention. Warfarin acts as a vitamin K antagonist and inhibits *γ*-carboxylation of coagulation factors II, V, IX, and X, protein C, and protein S [35]. It has a half-life of approximately 40 h, and this may be further increased in the elderly. In most patients with INR 2.0–3.0, the level falls to less than 1.5 within 96–115 h after the last warfarin dose [15]. However, if the steady state of INR is greater than 3.0, or the patient is elderly, more time is required for the level to fall to less than 1.5 [23]. 

Overanticoagulation can result from a multitude of factors. These include administration of inappropriately high warfarin doses, altered protein binding, decreased vitamin K intake, reduced vitamin K synthesis, and increased clearance of vitamin K-dependent clotting factors. Elderly hip fracture patients are frequently on other medications. Drugs that alter liver enzyme activity or compete with warfarin for protein binding will also affect the level of anticoagulation [36]. In addition, anticoagulant responses can be affected by various factors such as age, body weight, gender, and ethnicity [37].

Vitamin K therapy alone is inappropriate if immediate normalisation of the INR is required in hip fracture or actively bleeding patients. This is because the onset of action is 4–6 h after intravenous administration and at least 24 h after oral administration [34, 38]. High doses will not further shorten the time to anticoagulation reversal. However, high doses may lower INR more than is necessary and cause warfarin resistance that persists for up to 1 week [39]. Some authorities, such as the American College of Cardiology/American Heart Association, suggest avoidance of Vitamin K for patients with mechanical valves for fear of valve thrombosis [40]_._

Fresh frozen plasma can reverse anticoagulation immediately without causing any later resistance to warfarin or heparin [41]. However, its effect dissipates within 8–12 h and is optimally administered within 4 h of the procedure [42]. Risks include anaphylactoid reactions, alloimmunisation, excessive intravascular volume [42], and transmission of infection [21]. In addition, it also must be thawed before use, which can delay treatment.

Prothrombin complex concentrates (PCCs) contain high concentrations of the coagulation factors inactivated by warfarin, namely, factors II, VII, IX, and X, and can be rapidly administered. There are no studies evaluating the use of PCCs in hip fracture patients requiring anticoagulation reversal. A major advantage of PCCs over FFP is that smaller volumes of PCCs are required to reverse anticoagulation [37]. PCCs are also quicker to prepare than FFP [38]. The time required for INR correction was reported to be five times more rapid with PCCs than FFP [43]. One important consideration is the association of FFP with a risk of transfusion-related acute lung injury, which has not been documented as a safety concern with PCCs [44]. However, the primary safety concern with PCCs has been their association with thrombogenic events such as stroke, myocardial infarction, pulmonary embolism, disseminated intravascular coagulation, and deep vein thrombosis [45]. The majority of hip fracture patients do not require immediate surgery. Therefore, the risks of administering PCCs to reverse anticoagulation generally outweigh the benefits. Surgeons should, however, be aware of the availability of PCCs for rapid anticoagulation reversal.

There are no guidelines for perioperative bridging therapy for patients on long-term warfarin requiring surgery. However, it is advocated that most patients except those at the highest risk of thromboembolism, while off warfarin, do not need bridging therapy. High-risk patients (e.g., with mechanical prosthetic valves) are recommended to receive unfractionated heparin whilst the INR is subtherapeutic [46]. Discussion with the haematology and cardiology departments is mandatory in this group of patients in order to carefully manage their anticoagulation.

There were limitations of our study. Patients who were not local to our area could not be included. This was due to the fact that GP anticoagulation records were not available. Therefore, not all warfarinised patients undergoing hip fracture surgery in our unit were included. As previously mentioned, not all surgeons and anaesthetists in our unit required the INR to be strictly less than 1.5 before undertaking the procedure. Another limitation is the lack of randomisation and the retrospective analysis which make this study liable for selection bias. 

Previous studies have reported similar experience with hip fracture patients on warfarin [9, 13, 14, 17]. Al-Rashid et al. reviewed the literature and recommended that hip fracture patients should be stratified into high- or low-risk categories based on clinical indication for warfarin and comorbidities [13]. Low-risk patients can be given intravenous or oral Vitamin K until INR is below 1.5. For high-risk patients requiring surgery immediately or on the same day, fresh frozen plasma should be considered. Bridging therapy with low molecular heparin should be used for high risk patients for the duration of the subtherapeutic INR. 

## 5. Conclusion

This study confirms the variation in practice for management of hip fracture patients on warfarin needing semi-urgent surgery. Reversal of warfarin anticoagulation facilitated earlier surgery. Patients on warfarin admitted with hip fractures should have their anticoagulation reversed to avoid significant delays in surgery. Further prospective studies are needed to assess the best management of warfarin anticoagulation in acute preoperative admissions through a combined effort from surgeons, haematologists, and cardiologists. A national policy should be developed for reversing warfarin anticoagulation in patients with hip fractures requiring surgery. Current evidence favours the use of oral vitamin K for anticoagulation reversal in hip fracture patients.

##  Conflicts of Interest

The authors declare that there are no conflict of interest.

## References

[1] Marzolini M, Wynne HA (2003). Should patients manage their own oral anticoagulation therapy?. *Reviews in Clinical Gerontology*.

[2] Sudlow M, Thomson R, Thwaites B, Rodgers H, Kenny RA (1998). Prevalence of atrial fibrillation and eligibility for anticoagulants in the community. *The Lancet*.

[3] Klein M, Velan GJ (2006). The timing of surgery for hip fracture: the case for early repair. *Israel Medical Association Journal*.

[4] Moran CG, Wenn RT, Sikand M, Taylor AM (2005). Early mortality after hip fracture: is delay before surgery important?. *Journal of Bone and Joint Surgery. American*.

[5] Perez JV, Warwick DJ, Case CP, Bannister GC (1995). Death after proximal femoral fracture: an autopsy study. *Injury*.

[6] Scottish Intercollegiate Guidelines Network (SIGN) (2002). Prevention and management of hip fracture in older people; a national clinical guideline. *SIGN Publication*.

[7] Shiga T, Wajima Z, Ohe Y (2008). Is operative delay associated with increased mortality of hip fracture patients? Systematic review, meta-analysis, and meta-regression. *Canadian Journal of Anesthesia*.

[8] Sircar P, Godkar D, Mahgerefteh S, Chambers K, Niranjan S, Cucco R (2007). Morbidity and mortality among patients with hip fractures surgically repaired within and after 48 hours. *American Journal of Therapeutics*.

[9] Tharmarajah P, Pusey J, Keeling D, Willett K (2007). Efficacy of warfarin reversal in orthopedic trauma surgery patients. *Journal of Orthopaedic Trauma*.

[10] Dahl OE, Gudmundsen TE, Haukeland L (2000). Late occurring clinical deep vein thrombosis in joint-operated patients. *Acta Orthopaedica Scandinavica*.

[11] Frostick SP (2000). Death after joint replacement. *Haemostasis*.

[12] Gallus AS, Baker RI, Chong BH, Ockelford PA, Street AM (2000). Consensus guidelines for warfarin therapy: recommendations from the Australasian society of thrombosis and haemostasis. *Medical Journal of Australia*.

[13] Al-Rashid M, Parker MJ (2005). Anticoagulation management in hip fracture patients on warfarin. *Injury*.

[14] Bansal R, Watson DK (2005). Surgical delay in acute admissions on warfarin: are we doing enough?. *International Journal of Clinical Practice*.

[15] White RH, McKittrick T, Hutchinson R, Twitchell J (1995). Temporary discontinuation of warfarin therapy: changes in the international normalized ratio. *Annals of Internal Medicine*.

[16] Starks I, Cooke S, Docker C, Raine A (2009). Warfarinized patients with proximal femoral fractures: survey of UK clinical practice. *European Journal of Trauma and Emergency Surgery*.

[17] Verma R, Tayton E, Birch B (2008). Does oral vitamin K given in A&E help expedite surgery in fracture neck of femur patients on warfarin for atrial fibrillation?. *Injury Extra*.

[18] Laliberté R, Chakrabarti S, Brodeur J (1976). The influence of fasting and stress on the response of rats to warfarin. *Journal of Pharmacology and Experimental Therapeutics*.

[19] Despotis GJ, Filos KS, Zoys TN, Hogue CW, Spitznagel E, Lappas DG (1996). Factors associated with excessive postoperative blood loss and hemostatic transfusion requirements: a multivariate analysis in cardiac surgical patients. *Anesthesia and Analgesia*.

[20] Landefeld CS, Cook EF, Flatley M, Weisberg M, Goldman L (1987). Identification and preliminary validation of predictors of major bleeding in hospitalized patients starting anticoagulant therapy. *American Journal of Medicine*.

[21] Travis S, Wray R, Harrison K (1989). Perioperative anticoagulant control. *British Journal of Surgery*.

[22] Baglin TP, Keeling DM, Watson HG (2006). Guidelines on oral anticoagulation (warfarin): third edition—2005 update. *British Journal of Haematology*.

[23] Jaffer AK, Brotman DJ, Chukwumerije N (2003). When patients on warfarin need surgery. *Cleveland Clinic Journal of Medicine*.

[24] Spandorfer J (2001). The management of anticoagulation before and after procedures. *Medical Clinics of North America*.

[25] Hynson JM, Katz JA, Bueff HU (1996). Epidural hematoma associated with enoxaparin. *Anesthesia and Analgesia*.

[26] Porterfield WR, Wu CL (1997). Epidural hematoma in an ambulatory surgical patient. *Journal of Clinical Anesthesia*.

[27] Vandermeulen EP, Van Aken H, Vermylen J (1994). Anticoagulants and spinal-epidural anesthesia. *Anesthesia and Analgesia*.

[28] Douketis JD (2002). Perioperative anticoagulation management in patients who are receiving oral anticoagulant therapy: a practical guide for clinicians. *Thrombosis Research*.

[29] Baker M, Ollivere AJ (2008). Low-dose oral vitamin K is a safe and effective method for controlled anticoagulation reversal in orthopaedic trauma patients. *Injury Extra*.

[30] Brooksbank A, Sckhtivel S, Rickhuss P (2004). The incidence and management of hip fracture patients admitted on warfarin, a prospective study. *The Journal of Bone and Joint Surgery. British*.

[31] Kirsch MJ, Vrabec GA, Marley RA, Salvator AE, Muakkassa FF (2004). Preinjury warfarin and geriatric orthopedic trauma patients: a case-matched study. *Journal of Trauma-Injury, Infection and Critical Care*.

[32] Baker P, Gleghorn A, Tripp T, Paddon K, Eagleton H, Keeling D (2006). Reversal of asymptomatic over-anticoagulation by orally administered vitamin K. *British Journal of Haematology*.

[33] Hanslik T, Prinseau J (2004). The use of vitamin K in patients on anticoagulant therapy: a practical guide. *American Journal of Cardiovascular Drugs*.

[34] Ansell J, Hirsh J, Hylek E, Jacobson A, Crowther M, Palareti G (2008). Pharmacology and management of the vitamin K antagonists: American College of Chest Physicians Evidence-Based Clinical Practice Guidelines (8th Edition). *Chest*.

[35] Levy JH, Tanaka KA, Dietrich W (2008). Perioperative hemostatic management of patients treated with vitamin K antagonists. *Anesthesiology*.

[36] (1998). Haemostasis and Thrombosis Task Force for the British Committee for Standards in Haematology. Guidelines on oral anticoagulation: third edition. *British Journal of Haematology*.

[37] Hanley JP (2004). Warfarin reversal. *Journal of Clinical Pathology*.

[38] Makris M, Watson HG (2001). The management of coumarin-induced over-anticoagulation. *British Journal of Haematology*.

[39] Ansell J, Hirsh J, Dalen J (2001). Managing oral anticoagulant therapy. *Chest*.

[40] Bonow RO, Carabello B, de Leon AC (1998). Guidelines for the management of patients with valvular heart disease: executive summary: a report of the American College of Cardiology/American Heart Association task force on practice guidelines (Committee on Management of Patients With Valvular Heart Disease). *Circulation*.

[41] Heit JA (2001). Perioperative management of the chronically anticoagulated patient. *Journal of Thrombosis and Thrombolysis*.

[42] (September 1984). Fresh frozen plasma: indications and risks. *NIH Consensus Statement*.

[43] Fredriksson K, Norrving B, Stromblad LG (1992). Emergency reversal of anticoagulation after intracerebral hemorrhage. *Stroke*.

[44] Bux J (2005). Transfusion-related acute lung injury (TRALI): a serious adverse event of blood transfusion. *Vox Sanguinis*.

[45] Köhler M, Hellstern P, Lechler E, Überfuhr P, Müller-Berghaus G (1998). Thromboembolic complications associated with the use of prothrombin complex and factor IX concentrates. *Thrombosis and Haemostasis*.

[46] Jafri SM (2004). Periprocedural thromboprophylaxis in patients receiving chronic anticoagulation therapy. *American Heart Journal*.

